# Ni-Doped Ordered Nanoporous Carbon Prepared from Chestnut Wood Tannins for the Removal and Photocatalytic Degradation of Methylene Blue

**DOI:** 10.3390/nano12101625

**Published:** 2022-05-10

**Authors:** Ruby Bello, Elena Rodríguez-Aguado, Victoria A. Smith, Dmitry Grachev, Enrique Rodríguez Castellón, Svetlana Bashkova

**Affiliations:** 1 Department of Chemistry, Biochemistry and Physics, Fairleigh Dickinson University, Madison, NJ 07940, USA; ruby_b@student.fdu.edu (R.B.); james_d@student.fdu.edu (V.A.S.); dmi_gra5@student.fdu.edu (D.G.); 2 Departamento de Química Inorgánica, Facultad de Ciencias, Universidad de Málaga, Campus de Teatinos, 29071 Málaga, Spain; aguadoelena5@gmail.com (E.R.-A.); castellon@uma.es (E.R.C.)

**Keywords:** ordered nanoporous carbon, water purification, photocatalysis, visible light, nickel, soft-templating, methylene blue, adsorption

## Abstract

In this work, Ni-doped ordered nanoporous carbon was prepared by a simple and green one-pot solvent evaporation induced self-assembly process, where chestnut wood tannins were used as a precursor, Pluronic^®^ F-127 as a soft template, and Ni^2+^ as a crosslinking agent and catalytic component. The prepared carbon exhibited a 2D hexagonally ordered nanorod array mesoporous structure with an average pore diameter of ~5 nm. Nickel was found to be present on the surface of nanoporous carbon in the form of nickel oxide, nickel hydroxide, and metallic nickel. Nickel nanoparticles, with an average size of 13.1 nm, were well dispersed on the carbon surface. The synthesized carbon was then tested for the removal of methylene blue under different conditions. It was found that the amount of methylene blue removed increased with increasing pH and concentration of carbon but decreased with increasing concentration of methylene blue. Furthermore, photocatalytic tests carried out under visible light illumination showed that purple light had the greatest effect on the methylene blue adsorption/degradation, with the maximum percent degradation achieved at ~4 h illumination time, and that the percent degradation at lower concentrations of methylene blue was much higher than that at higher concentrations. The adsorption/degradation process exhibited pseudo second-order kinetics and strong initial adsorption, and the prepared carbon showed high magnetic properties and good recyclability.

## 1. Introduction

Ordered mesoporous carbons (OMCs) have recently attracted the attention of many researchers due to their unique properties and their variety of applications. OMCs are known for their uniform mesoporosity, high surface area, large pore volume, and chemical inertness. These properties enable the application of OMCs in catalysis, separation, energy storage, and environmental remediation [1,2,3,4,5,6,7,8,9,10,11,12]. The wide uniform pores are highly suitable for the separation of large organic molecules and serve as channels for the fast mass transfer of these molecules to the active sites. Two templating techniques are commonly used for the preparation of OMCs, hard-templating and soft-templating. Hard-templating, or nanocasting, is widely applied, as it permits precise control of pore size and shape by utilizing a template like mesoporous silica [3,4,5,7,8,11,12]. However, the disadvantages of this technique include the use of hazardous chemicals such as hydrofluoric acid (HF) and/or sodium hydroxide (NaOH) needed to remove the silica template, and the process of templating is considered time-consuming and costly. As for the soft-templating process, it can be quite complex and must comply with several criteria. Here, the templating strategy is based on supramolecular self-assembly via interaction between the amphiphilic nonionic copolymer and the functional groups of the carbon precursor [2,9,10,12,13,14], where successful formation of a micelle largely depends on the presence of reactive precursor groups necessary for polymerization and crosslinking, as well as on the presence of the rigid polymeric backbone to prevent the collapse of the mesopore structure.

Some commonly used carbon precursors such as phenols, resorcinol and phloroglucinol [12,13,14] are not sustainable. Moreover, the key crosslinking reaction in micelle formation is limited to the acid- or base-catalyzed polycondensation of toxic phenol-formaldehyde [12,13,14]. Thus, overcoming the drawbacks of hard-templating and soft-templating techniques has recently become the focus of many research groups. Some natural renewable biomass resources, like tannins and lignin, have been explored as carbon precursors for OMCs [2,15,16,17,18]. Sanchez-Sanchez et al. [2] showed the successful formation of OMCs with more developed porosities, more ordered mesostructures, and higher nanotexture ordering from mimosa tannins and Pluronic^®^ F-127 under mild conditions (pH 3, 30 °C) without the use of formaldehyde. Herou et al. [18] reported the synthesis of sustainable OMCs with highly ordered pore structure from lignin, using evaporation-induced self-assembly (EISA) method, where they replaced half of the phloroglucinol with hard-wood organosolv lignin, and replaced the formaldehyde with glyoxal as a crosslinker. Likewise, Song et al. [17] used lignin (pre-crosslinked) as a precursor but employed nano-sized magnesium oxide (MgO) and Pluronic^®^ F-127 as templates, where MgO was found to control porosity, which was significantly enlarged compared to a single-template route. In all the above cases, OMCs were efficiently applied as supercapacitors for energy storage. In addition, the applicability of OMCs can further be enhanced by loading active metals such as nickel and zinc on their surface [19,20,21,22,23,24,25,26]. Common doping methods include wet impregnation and coprecipitation, but both require complex operation procedure and may result in metal leaching [20].

In this work, we use the simple EISA technique [20] with chestnut wood tannins as a carbon precursor and nickel ions (Ni^2+^) as a crosslinker to prepare embedded ordered nanoporous carbons. In the synthesis process, Ni^2+^ ions are used to crosslink the micelle composites of tannins and Pluronic^®^ F-127 by coordinative interaction with the hydroxyl groups of the tannins. Thus, Tang et al. [20] described the successful preparation of Ni-doped mesoporous carbons by a one-pot EISA process using gallic acid as the carbon precursor, Pluronic^®^ F-127 as the soft template, and Ni^2+^ as the crosslinker and catalytically active ingredient for quantitative hydrogenation of furfural to furfuryl alcohol. In addition, Ni-containing OMCs have been reported to possess magnetic properties, which are highly desirable, specifically in the area of adsorption/separation. Furthermore, Ni-doped OMCs may also exhibit photocatalytic activity and thus will be tested here for photocatalytic removal of methylene blue (MB) under visible light. Methylene blue is a cationic azo dye found in wastewater, which is a significant source of water pollution from the textile, paper, and printing industries [27]. Due to its toxicity, and its slow and complex biodegradation with the possible release of carcinogenic products, MB may have various detrimental effects on human and aquatic life when released into the environment [27]. OMCs are expected to be well suited for the removal of bulky MB molecules from water, due to their high specific surface area and pore volume, homogenous distribution of pores of uniform size, and high structural stability. Moreover, nickel-containing OMCs may potentially enhance removal of MB by its catalytic degradation under the UV or visible light illumination.

Although titanium dioxide is widely reported to be one of the best photocatalysts for environmental purification due to its great photo-oxidative properties [28,29,30,31,32,33], nickel oxide has also proved to be very efficient in this extent [34,35,36]. Thus, the latter is known to be a p-type semiconductor with a band gap of 3.5 eV that is stable, non-toxic, and has high photocatalytic activity [34,35,36]. Overall, the process of photocatalytic degradation appears to be very attractive, as it involves the production of highly active radicals that can unsystematically and rapidly decompose a wide range of pollutants, resulting in the formation of harmless products such as carbon dioxide and water [26,28,29,30,31,32,33,34,35,36]. Several mechanisms for the photocatalytic degradation of dyes have been suggested in literature [26,29,30,31,32,33,36]. One, where the dye adsorbed on the surface of the semiconductor photocatalyst absorbs a photon and excites an electron to the conduction band of the photocatalyst, followed by the reaction of this activated electron with the oxygen molecule to generate the reduced superoxide radical anion that further degrades the dye molecule to non-toxic products [30,36]. In the other, the direct photoexcitation of the semiconductor takes place first, forming an electron–hole pair on the surface of the catalyst, followed by the reaction between the hole and the dye, or the hole and water or hydroxide ions to form the oxidized product, hydroxyl radicals, causing dye degradation [26,29,33,36]. Furthermore, if photocatalytic oxidation is combined with conventional purification methods like adsorption, coagulation, and biological purification, it could become one of the most efficient ways of purifying industrial waters from organic water pollutants such as synthetic dyes.

The novelty of this work is in the application of Ni-doped nanoporous carbons prepared by simple and green one-pot synthesis in the removal of MB. Although a vast variety of samples have been successfully tested for MB removal and photodegradation, there is no published work on MB removal by Ni-OMCs prepared by one-pot evaporation induced self-assembly method (EISA). The advantages of this method include its facility, effectiveness, and speed, which in combination with a green chemistry approach presents an attractive alternative to the other methods of synthesis. To prepare OMCs, sustainable chestnut wood tannins are used as a carbon precursor, Pluronic^®^ F-127 as a soft template, and Ni^2+^ as a crosslinking agent that successfully replaces the carcinogenic formaldehyde commonly used in the preparation of OMCs. These OMCs are expected to exhibit high stability due to the space confinement effects of the carbon skeleton on the doped nickel particles through coordinative interactions between tannins and Ni^2+^ ions [20]. Moreover, all photocatalytic studies are conducted in the visible light conditions to further support the sustainable approach.

## 2. Materials and Methods

### 2.1. Preparation of the Ni-OMC Sample

Chestnut wood tannins were purchased from LD Carlson Company in Kent, OH, USA. Pluronic^®^ F-127 ((C_3_H_6_O·C_2_H_4_O)_x_), Ni(II) nitrate hexahydrate (H_12_N_2_NiO_12_), pure ethyl alcohol (C_2_H_6_O) and 1.0 N hydrochloric acid (HCl) were purchased from Sigma-Aldrich, St. Louis, MO, USA. To prepare Ni-OMC samples, slightly modified one-pot EISA method described by Tang et al. [20] was used. Here, 2 g of chestnut wood tannins with 40 mL of ethanol and 1 mL of 1.0 N HCl was added to one beaker, and 2 g of Pluronic^®^ F-127 with 40 mL of ethanol was added to another beaker, followed by mixing the content of the two beakers and allowing this mixture to stir for about 30 min. After this, 1 g of Ni(II) nitrate hexahydrate was dissolved in 10 mL of ethanol, added to the Pluronic^®^ F-127-tannin mixture, and allowed to stir for 30 min. This mixture (1:1:0.5 tannin-Pluronic^®^ F-127-Ni by weight) was then poured into evaporating dish and left to evaporate overnight, followed by drying in the oven at 105 °C overnight. Finally, the dried mixture was carbonized in a horizontal tube furnace (Lindberg Blue M 1100C, Thermo Scientific, Waltham, MA, USA), using the following procedure. The sample was placed in a carbonization boat inside the furnace, where it was heated in a stepwise manner under the flow of nitrogen at 50 mL/min. First, the sample was heated to 400 °C at a heating rate of 1 °C/min and held there for four h. Second, it was heated to 900 °C at a rate of 2 °C/min and held there for 1 h. Lastly, the sample was cooled to ambient temperature at a rate of 10 °C/min. The resulting carbon was named TPNi, where the letters stand for tannins, Pluronic^®^ F-127, and nickel.

### 2.2. Batch Adsorption Studies

Methylene blue, MB (C_16_H_18_ClN_3_S·xH_2_O), was purchased from Sigma-Aldrich, St. Louis, MO, USA, hydrochloric acid (HCl) and sodium hydroxide (NaOH) were purchased from Fisher Scientific, Pittsburgh, PA, USA.

To study the effect of the initial concentration of MB on the amount of MB removed by TPNi, solutions of 40, 50 and 60 mg/L of MB were prepared. For a typical batch adsorption test, 25 mL of MB solution of each concentration were mixed with 15 mg of TPNi, previously sieved to 250 μm particle size, and shaken for 24 h at 175 rpm on a compact digital mini rotator (Thermo Scientific, Waltham, MA, USA). These mixtures were then filtered through 12.5 cm filter paper, and the absorbance of each filtrate was measured with the Olis 8453 UV–Visible Spectrophotometer (Olis Inc., Athens, GA, USA) at ~665 nm.

To study the effect of pH on the amount of MB removed by TPNi, a solution of 60 mg/L of MB was prepared and separated into three aliquots, two of which were adjusted to pH 3 and 9, using 0.1 N HCl and 0.1 N NaOH, respectively, while the third was left as is; the pH of each solution was measured using Piccolo ATC pH tester (Hanna Instruments, Woonsocket, RI, USA). Then, 25 mL of each aliquot were mixed with 15 mg of sieved TPNi, followed by the same steps as in the study of MB concentration.

To investigate the effect of the sample dose on the amount of MB removed by TPNi, a solution of 60 mg/L of MB was prepared and 25 mL aliquots of this solution were mixed with 15, 20 and 25 mg of the sieved TPNi, followed by the same procedural steps as described above.

To study the impact of the tannin−Pluronic^®^ F-127−Ni ratio on the amount of MB removed, the initial 1:1:0.5 ratio was adjusted to 1:2:0.5 and 2:1:0.5, varying the mass ratio between tannin and Pluronic^®^ F-127 but keeping the mass of Ni the same. Then, each ratio of TPNi sample (15 mg) was mixed with 25 mL of 60 ppm MB, followed by the same steps as before.

In each case, the concentrations of the filtrate solutions were calculated from the absorbance values using external standard calibration. The amount of MB removed at time *t*, $q_{t}$ (mg/g), and the removal efficiency of carbon (%) were calculated using Equations (1) and (2), respectively.

(1) $$q_{t}=\left(c_{0}-c_{t}\right)V/m$$

(2) $$Removal\;efficiency\;\%=\left(c_{0}-c_{t}\right)/c_{0}\times 100$$

where $c_{0}$ is the initial concentration of MB, $c_{t}$ is the concentration of MB at time *t*, V is the volume of MB solution in L, and m is the mass of TPNi sample in g.

### 2.3. Photocatalytic Studies

The photocatalytic activity of the TPNi sample was studied by exposing the mixtures of TPNi and MB to visible light, green (Kessil KSPR 160 L-525 nm LED Photoredox Light, Kessil, Richmond, CA, USA) and purple (Kessil KSPR 160 L-390 nm LED Photoredox Light, Kessil, Richmond, CA, USA), for different time intervals. In a typical run, a solution of 60 mg/L of MB was prepared and 25 mL of this solution were mixed with 15 mg of TPNi. The mixture was then shaken for 2, 4, 6, and 8 h at 175 rpm on a digital mini rotator under the green light of maximum intensity. At the end of each time interval, the mixture was filtered, and the absorbance of each filtrate was measured at ~665 nm. These tests were also conducted under purple light and in the dark and were repeated for multiple batches of TPNi. The amount of MB removed in each case was calculated according to Equation (1), and the amount of MB degraded upon light illumination was calculated from the amount removed under light, $q_{light}$, and the amount removed in the dark, $q_{dark}$ (Equation (3)).

(3) $$Degradation\;\%=\left(q_{light}-q_{dark}\right)/q_{dark}\times 100$$

### 2.4. Kinetics Studies

To study the kinetics of the adsorption process, 15 mg of the sieved TPNi samples was combined with 25 mL of 60 mg/L MB solution and shaken at 175 rpm. Aliquots were collected every 30 min within the first hour, followed by collecting an aliquot at 1, 2, 3, 4, 5, 8, 16 and 20 h. Solutions were then filtered, and absorbance values were measured at ~665 nm. Kinetics experiments were conducted in the dark as well as under green and purple light illumination. The kinetics data were analyzed using three different kinetics models.

For the pseudo first-order model, the Lagergren’s equation (Equation (4)) [37] was used in its linearized form (Equation (5)):

(4) $$q_{t}=q_{e}\left(1-e^{-k_{1}t}\right)$$

(5) $$\ln\left(q_{e}-q_{t}\right)=-k_{1}t+\ln q_{e}$$

where $k_{1}$ (1/min) is the psuedo first-order rate constant, *t* (min) is the contact time, and $q_{t}$ (mg/g) is the amount of MB adsorbed at time *t* (min), and $q_{e}$ (mg/g) is the amount of MB adsorbed at equilibrium (min).

If plotting $\ln\left(q_{e}-q_{t}\right)$ vs. *t* produces a straight line, the pseudo first-order model applies, and $k_{1}$ is found from the negative slope of the line, and $q_{e}$ is calculated by taking the exponent of the *y*-intercept.

For the pseudo second-order model, the equation proposed by Ho and McKay (Equation 6) [38] was applied in its linearized form (Equation (7)):

(6) $$q_{t}=\frac{q_{e}^{2}k_{2}t}{1+q_{e}k_{2}t}$$

(7) $$\frac{t}{q_{t}}=\left(\frac{1}{q_{e}}\right)t+\frac{1}{q_{e}^{2}k_{2}}$$

where $k_{2}$ (g/mg min) is the pseudo second-order rate constant.

If plotting $\frac{t}{q_{t}}\;\mathrm{vs}.\;t$ produces a straight line, the pseudo second-order model applies, and $k_{2}$ is found as the ratio of the squared slope of the line to the *y*-intercept, and $q_{e}$ is calculated as the reciprocal of the slope.

For intra-particle diffusion, the model proposed by Weber and Morris [39] was used (Equation (8)):

(8) $$q_{t}=k_{i}t^{1/2}+C$$

where $k_{i}$ is the intra-particle diffusion rate constant (g/mg min^1/2^) and C (mg/g) is the intercept of the plot, which reflects the thickness of the boundary layer; the larger is the value, the greater is the boundary layer effect.

If plotting $q_{t}\;\mathrm{vs}.\;t^{1/2}$ produces a straight line, the intra-particle diffusion model applies, and $k_{i}$ is equal to the slope of the linear line, and C, to the intercept.

### 2.5. Recyclability Studies

For each run, 15 mg of the sieved TPNi were mixed with 25 mL of 60 mg/L MB solution and left on the shaker at 175 rpm for 20 h either in the dark or under purple light. After this, the carbon sample was separated from the MB solution with a magnet, the MB solution was decanted and its absorbance measured at ~665 nm, while the carbon sample was washed repeatedly with ethanol and water until clear. The carbon sample was then tested three more times, following the same procedure.

### 2.6. X-ray Photoelectron Spectroscopy (XPS)

XPS studies were performed on a Physical Electronics Instruments spectrometer (PHI Versa Probe II), Chanhassen, MN, USA, using monochromatic Al Kα radiation (25.1 W, 15 kV, 1486.6 eV) and a dual beam charge neutralizer for analyzing the core-level signals of the elements of interest with a hemispherical multichannel detector. The activated carbon sample spectra were recorded with a constant pass energy value at 29.35 eV and a beam diameter of 100 µm. Energy scale was calibrated using Cu 2*p*_3/2_, Ag 3*d*_5/2_, and Au 4*f*_7/2_ photoelectron lines at 932.7, 368.2, and 83.95 eV, respectively. The X-ray photoelectron spectra obtained were analyzed using PHI SmartSoft software and processed using MultiPak 9.6.0.15 package. The binding energy values were referenced to C1*s* signal at 284.5 eV. Shirley-type background and Gauss–Lorentz curves were used to determine the binding energies. Atomic concentration percentages of the characteristic elements were determined considering the corresponding area sensitivity factor for the different measured spectral regions.

### 2.7. X-ray Diffraction (XRD)

Laboratory X-ray powder diffraction (XRPD) patterns were collected on a Malvern PANalytical EMPYREAN automated diffractometer, Malvern UK. Powder patterns were recorded in Bragg-Brentano reflection configuration by using the PIXcel 3D detector with a step size of 0.017° (2θ). The powder patterns were recorded between 5 and 80 in 2θ with a total measuring time of 30 min.

### 2.8. Nitrogen Sorption

Nitrogen adsorption/desorption experiments were performed at −196 °C to determine specific surface area ($S_{BET}$) from multi-point BET method [40], total pore volume ($V_{tot}$), at P/P_0_ = 0.94, from single point adsorption, mircopore volume ($V_{mic}$) from t-plot analysis, pore size distributions and average pore diameter (w) from Barrett–Joyner–Halenda (BJH) method [41], using desorption branches of the isotherms. The samples were outgassed at 150 °C and 10^−4^ mbar overnight using an automatic ASAP 2020 system (Micromeritics, Norcross, GA, USA).

### 2.9. Transmission Electron Microscopy (TEM)

Distribution, morphology, and size of the nanoparticles were studied by high-resolution transmission electron microscopy (HRTEM) using a TALOS F200x instrument, Thermo Scientific, Waltham, MA, USA. Samples were suspended in ethanol and treated by ultrasound for 15 min. A drop of the suspension was deposited on a quantifoil holey carbon film supported by a copper grid and dried before analysis. Image J software was used to calculate the average particle size distribution.

## 3. Results

### 3.1. Textural and Structural Characterization

**Figure 1:**
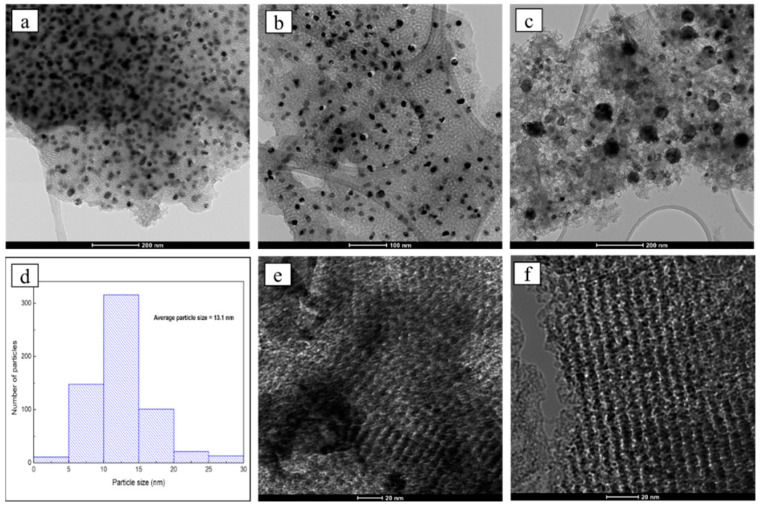
High-resolution TEM images of TPNi (**a**–**c**,**e**,**f**) and average nickel particle size distribution for TPNi (**d**).

The distribution, morphology and size of the nickel nanoparticles were studied using high-resolution transmission electron microscopy (HRTEM). In general, the micrographs corresponding to the TPNi material showed well dispersed nickel nanoparticles with even shapes and sizes (Figure 1a,b). However, the coexistence of larger and smaller particles with different shapes was also noticeable, as well as the existence of areas where the particles are not so uniformly dispersed and are agglomerated (Figure 1c). Therefore, the average particle size distribution of nickel nanoparticles has been measured by statistical evaluation of the particle sizes from selected regions of different micrographs. To reflect reality more accurately, more than 600 particles were measured, since it is known that the number of particles needed for high accuracy estimates of the average diameter depends on the spread of the particle size distribution [42]. Since the shape of these nanoparticles is not always completely spherical and, depending on how they sit on the substrate, they my appear to be less spherical, elongated or triangular, irregularly shaped particles were included in the measurement. However, overlapping particles resting on top of each other were excluded [43]. As a result, the material presents a broad nickel particle size distribution with an average particle size of 13.1 nm, as shown in the histogram in Figure 1d.

Regarding the morphology of the carbonaceous support, the mesoporous structure of the support was clearly observed. Herein, the regular strip-shaped channels were clearly noted from the perpendicular channel direction (Figure 1e), and the hexagonally arranged pore structure was observed along the channel direction (Figure 1f), indicating the 2D hexagonally ordered nanorod array mesoporous structure [44].

**Figure 2:**
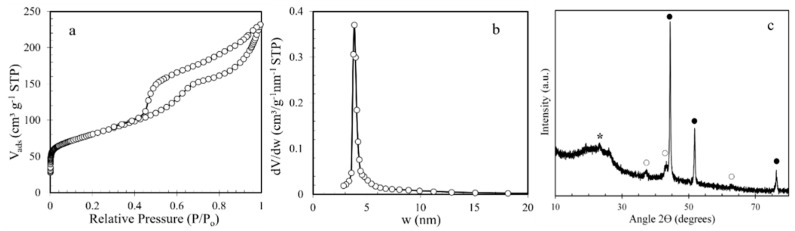
(**a**) Nitrogen adsorption−desorption isotherm, (**b**) PSD of TPNi (w—pore width in nm), and (**c**) XRD profile of TPNi (* amorphous carbon, ○ nickel oxide, ● metallic nickel).

The textural parameters of TPNi and its carbonization yield are shown in Table 1. The yield was 22.0 ± 1.9%, and the surface area and mesopore volume were 284 m^2^/g and 0.304 cm^3^/g, respectively. As seen in Figure 2a, TPNi exhibited a type IV isotherm with an H-1-type hysteresis loop at P/P_0_ ~ 0.4−1.0 [19], characteristic of mesoporous materials with interconnected cylindrical pores and narrow pore size distributions [2,19,20,45]. The mesoporous nature of TPNi was further confirmed by its pore size distribution (PSD) pattern (Figure 2b), which is very narrow with a peak value of ~3.8 nm, and its negligible volume of micropores (Table 1). These results are in agreement with the textural characteristics of the Ni-containing OMC samples obtained by Tang et al. [20], from whom the synthesis procedure of TPNi was adopted. In our case, we used a two-step carbonization procedure, unlike Tang and co-workers [20], who used a one-step procedure with slow heating to 700 °C. The two-step carbonization was chosen based on the results obtained by Sanchez-Sanchez et al. [2], where they found that an additional isothermal step at 400 °C results in a better-preserved carbon mesostructure.

The crystalline structure of TPNi was studied by XRD in the range of 2 θ Bragg angles of 5 to 80°. The XRD diffractogram of TPNi is presented in Figure 2c. Here, the broad diffraction peak between 15° and 30° indicates the presence of amorphous carbon [20,22,46]. Furthermore, the reflections at 37.3°, 43.3°, 44.4°, 51.8°, 62.8°, and 76.3° reveal the presence of different nickel species. Thus, the diffraction peaks at 37.3°, 43.3° and 62.8° can be assigned to nickel oxide structure [PDF card 04-006-6925], and the peaks at 44.4°, 51.8°, and 76.3° are characteristic of the face-centered cubic nickel [PDF card 04-016-6268]. The average particle size of nickel nanoparticles was calculated using Scherrer’s equation and was determined to be about 18 nm based on all the diffraction peaks between 37.3° and 76.3°. This is close to the results obtained by HRTEM, where average particle size of nickel nanoparticles was found to be 13.1 nm (Figure 1d). The discrepancy here arises from the fact that some diffraction peaks in Figure 2c appear small, and thus may not be accurately used for particle size determination. On the other hand, in HRTEM analysis, more than 600 nickel particles of different shapes have been measured, and therefore, these results represent a more accurate picture of nickel particle size distribution on the surface of TPNi.

### 3.2. Chemical Characterization

**Figure 3:**
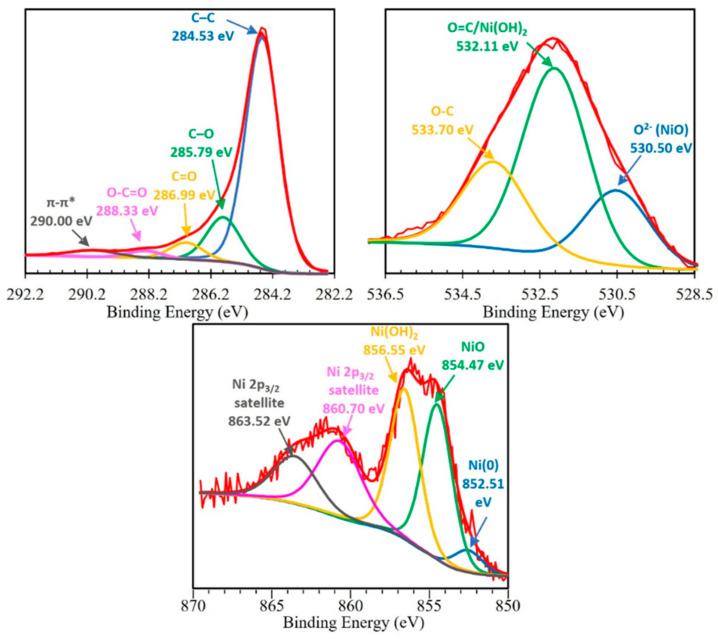
C1*s*, O1*s* and Ni 2*p* core-level spectra of TPNi.

The surface chemical composition of TPNi was studied by XPS, and the results of this study are collected in Table 2 and Figure 3. The surface concentration of oxygen was found to be 6.53 at. %, indicating the presence of some oxygen functionalities on the carbon surface. The deconvolution of C 1*s* spectrum revealed the nature of these functionalities, denoting the existence of C–C (284.5 eV), C–O (285.8 eV), C=O (287.0 eV), and O–C=O (288.3 eV) groups, and π−π* transitions (290.0 eV). Here, C–O groups (phenols and ethers [2,28,46]) are predominant, followed by C=O (carbonyls, quinones [2,28,44]), O–C=O (carboxylic [2,28,46]), and π−π* transitions (HOMO−LUMO transitions coming from the ring excited by the exciting photoelectrons for unsaturated carbon in aromatic rings [47]). Furthermore, the deconvolution of O 1*s* spectrum showed the presence of peaks at 530.5, 532.1 and 533.7 eV, which can be related to O^2-^ in nickel oxide [48], O=C in carbonyl and carboxylic groups [46], and O–C in phenols, ethers, and lactones [46], respectively. Interestingly, the amount of O=C functionalities in the O 1*s* spectrum is higher than the amount of O–C, which contradicts the results of the C 1*s* spectrum. This irregularity can be explained by the fact that the hydroxyl groups of Ni(OH)_2_ are located in the same binding energy region as O=C functionalities [49], therefore contributing to the overall at. % of O in this region. The nature of nickel functionalities was further explored by analyzing the Ni 2*p* spectrum. Herein, three species of Ni were identified: Ni^0^ at 852.5 eV [47,49,50,51], nickel oxide at 854.5 eV [47,49,50], and nickel hydroxide at 856.6 eV [47,49,50]; two additional peaks at 860.7 and 863.5 eV are assigned to satellite peaks of Ni 2*p*_3/2_ [47,48,49,50,51,52,53]. Evidently, most of the Ni is present on the surface of TPNi in the form of oxides and hydroxides, with only a small percent of it being reduced to the metal form.

### 3.3. Adsorption Studies

**Figure 4:**
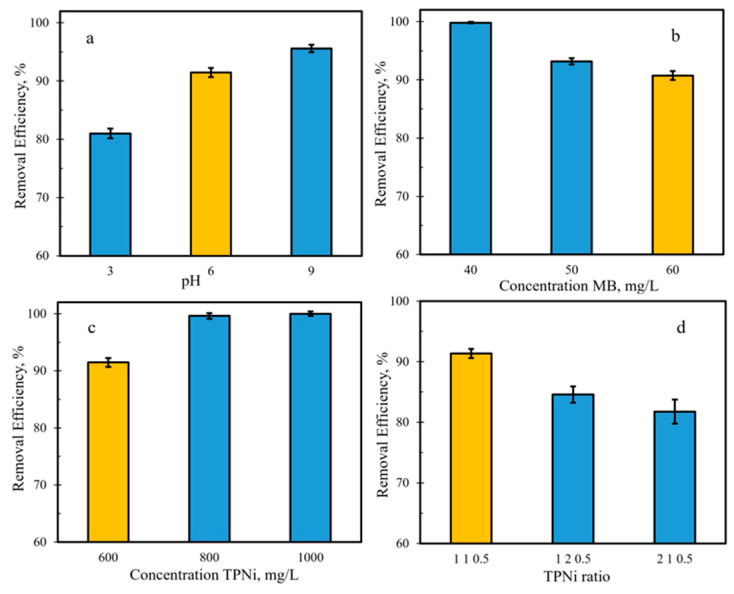
The effect of (**a**) pH, (**b**) concentration of MB, (**c**) concentration of TPNi carbon, and (**d**) TPNi mass ratio on the removal efficiency of MB by TPNi. Shown in yellow are the test conditions of pH 6, 60 mg/L MB, 600 mg/L TPNi, and 1:1:0.5 T:P:Ni ratio.

The effect of pH, initial MB concentration, carbon dose, and tannin−Pluronic^®^ F-127−Ni (T:P:Ni) ratio on the removal efficiency of MB (Equation (2)) by TPNi was investigated, and the average results of this study are shown in Figure 4, where the error bars represent standard errors for at least two batches of each TPNi sample, and every test repeated at least three times. The initial experimental conditions were chosen as: MB concentration of 60 ppm, TPNi concentration of 600 mg/L, pH of 6 (without adjustment), and tannin−Pluronic^®^ F-127−Ni ratio of 1:1:0.5. The carbon sample that corresponds to these conditions is shown in yellow in Figure 4. As seen in Figure 4a, the removal efficiency of MB by TPNi increases with increasing solution pH; this increase is more significant when going from pH 3 to pH 6 (~11%), and less significant when going from pH 6 to pH 9 (~4%). These results are in agreement with the literature data [27,33,54,55,56], where the increase in MB removal with increasing solution pH and surface basicity has been reported. Thus, the basic surface groups on carbon may attract MB molecules either by dipole–dipole interactions between negative oxygen sites and positive nitrogen of MB and/or by hydrogen bonding between the phenol groups of carbon and the aromatic rings of MB [27,54,56]. In the latter case, XPS detected a substantial number of phenols (C–O functionalities) on TPNi, as reported earlier in Table 2 and Figure 3.

The impact of the initial MB concentration on the removal efficiency of MB by TPNi was investigated by varying the concentration of MB from 40 to 60 mg/L. As shown in Figure 4b the removal efficiency decreased as the concentration of MB increased, with decreases of ~7% observed at a concentration of 40–50 mg/L and ~2%, in the range of 50–60 mg/L. The lower decrease in the latter case can be related to the saturation of active surface adsorption sites available for interaction with MB [56]; as the number of active sites on the surface approaches its maximum, the change in the removal efficiency of MB is no longer observed.

The effect of carbon dose on the removal efficiency of MB was also evaluated. The results in Figure 4c show that the removal efficiency of TPNi increased with the increase in carbon concentration from 600 to 1000 mg/L. It is also evident that the maximum removal efficiency value for a 60 mg/L MB solution was attained at around 800 mg/L of carbon, suggesting that at this point the number of MB molecules in solution that are available for the interaction with the active surface adsorption sites is approaching its limiting value.

Finally, the effect of the T:P:Ni ratio on the removal efficiency of MB was studied, and these results are presented in Figure 4d. Apparently, changing the T:P:Ni mass ratio from 1:1:0.5 to 1:2:0.5 or 2:1:0.5 caused the removal efficiency of carbon to go down, especially in the latter case (~10%). The proper ratio is highly important for successful self-assembly and micelle formation between tannins and Pluronic^®^ F-127, as it takes place via hydrogen-bonding between phenol groups on the former one and −O^−^ groups in the latter [2,14,15,18,20]. Furthermore, the addition of Ni^2+^ ions results in their coordination with the tannin phenol groups, which helps in crosslinking the tannins around the F-127 cluster [20,22]. The mass of Ni^2+^ was kept at 0.5 g, which was chosen based on the work of Tang et al. [20], who found that upon further increase of nickel(II) nitrate to 1.0 g, the ordered mesoporous structure disappeared. Moreover, our preliminary studies (not reported here) showed similar findings, that is, with increasing amount of nickel nitrate, a decrease in the removal efficiency of MB was observed.

### 3.4. Photocatalytic Studies

**Figure 5:**
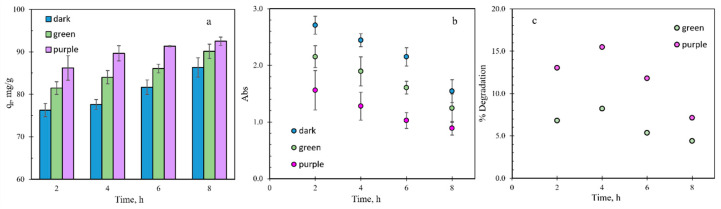
(**a**) The amount of MB removed over time, in the dark and under green and purple light, (**b**) MB filtrate absorbance over time, in the dark and under green and purple light, (**c**) % degradation over time under green and purple light.

To study the effect of visible light on the removal of MB, adsorption studies were performed in the dark, and then under green (525 nm) and purple light (390 nm) illumination. The results of this study are presented in Figure 5 for a 60 mg/L MB solution where measurements were made at 2, 4, 6, and 8 h illumination intervals. These results are the average of at least two batches of TPNi, and at least three repetitive runs for each test; error bars correspond to standard errors. In general, exposure to light increased the amount of MB removed, $q_{t}$ (Equation (1)). The irradiation intensity throughout the test was kept to maximum to ensure the success of the electron–hole recombination and the formation of free radicals [36]. As expected, the purple light had a greater impact on the amount of MB removed (Figure 5a–c), as it possesses more energy to promote electron–hole formation and to enhance the efficiency of the TPNi catalyst. As seen in Figure 5a–c, the rate of dye degradation slowed down with illumination time, which is especially visible for purple light. This can be related to the deactivation of active surface sites due to by-product deposition and a lower probability of reaction between the short chain aliphatic molecules and OH radicals [33,36]. Comparing the amount of MB removed in the dark, via adsorption, and the amount of MB removed under light, via adsorption and degradation, the percentage of MB removed by degradation was calculated (Equation (3)). Figure 5c shows that the maximum percent degradation for a 60 mg/L MB solution was reached at the illumination time of 4 h (~16% for purple and ~8% for green) and then decreased with time thereafter. It is also apparent that the % degradation for the purple light was consistently higher than that for green light.

**Figure 6:**
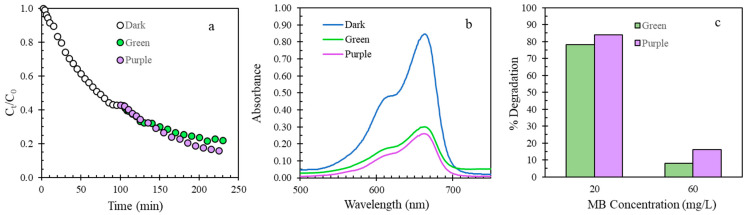
Adsorption and photodegradation under green and purple light of 20 mg/L MB by TPNi: (**a**) change in concentration of MB over time; (**b**) UV-Vis absorption spectra; (**c**) % degradation of 20 mg/L MB versus 60 mg/L MB.

Furthermore, to get a better perception of what is happening to MB during the photocatalytic test, a combined adsorption–photodegradation test was performed. First, adsorptive performance of TPNi was tested by collecting MB aliquots and measuring the absorbance every 2–10 min until the equilibrium state was reached. Then, photocatalytic performance was tested by exposing TPNi to light and measuring the absorbance of MB aliquots every 5–10 min. The results of this test for a 20 mg/L MB solution are shown in Figure 6a; the lower concentration of MB was chosen as it allows for a fast attainment of the equilibrium state during adsorption. As can be seen from Figure 6a, the concentration of MB in solution reaches an equilibrium after around 90 min of adsorption, but, upon exposure to light, the concentration of MB further decreases and approaches a limiting value at around 230 min. Apparently, more MB is being removed from solution under light, and as expected, the purple light has a greater effect on photocatalytic removal of MB (84% degradation) than the green light (78% degradation). It is also obvious that the removal of MB takes place by photodegradation rather than by desorption, as the absorbance of MB solution decreases with light (Figure 6b). Moreover, the products of MB photodegradation are adsorbed on the carbon surface, as no additional peaks were detected on the UV-Vis absorption spectra of MB after the photocatalytic test completion (Figure 6b). Interestingly, a much higher % degradation was achieved at 20 mg/L MB than at 60 mg/L MB (Figure 6c). This is most likely related to the fact that at higher concentrations of MB solution, there is significant competition between MB molecules and their photodegradation intermediates for the active adsorption sites, which pushes MB molecules back in the solution and deactivates the catalysis [57].

The photocatalytic degradation of MB in this work was compared to the results on similar types of materials reported in literature. As seen in Table 3, the range for MB% degradation on various types of materials varies from 40 to 100%, and our results fall within the higher end of this range, with 84% degradation of MB achieved at 120 min under purple light illumination. Although the results in Table 3 will strongly depend on multiple factor such as the concentration of MB, the concentration of the catalyst, the pH, the type and the intensity of the visible source used, the time of the degradation, the temperature, etc., the high percent degradation achieved in this work further supports the effectiveness of TPNi catalyst in the removal and photodegradation of MB under visible light conditions.

To better understand the kinetics of MB adsorption and degradation, kinetics data were collected as described in Section 2.4, and analyzed by applying psuedo first-order, pseudo second-order, and intra-particle diffusion models (Equations (4)–(8)). The results of this analysis are displayed in Table 4.

The adsorption/degradation of MB followed pseudo second-order kinetics for all testing conditions. Thus, the regression coefficients for the pseudo second-order model are close to 1, and the calculated $q_{e}$ values are very similar to those experimentally obtained (91.4 mg/g—dark, 93.6 mg/g—green, 94.7 mg/g—purple), with the best fit found for the purple light conditions. These results indicate that chemisorption is likely the rate-determining step, where sharing of electrons (valency forces) or exchange of electrons (covalent forces) between the adsorbent and adsorbate are involved [66]. Moreover, the increase in the magnitude of the rate constant from dark, to green, to purple conditions emphasizes the positive effect of visible light, specifically of shorter wavelength, on the rate of adsorption/degradation of MB.

**Figure 7:**
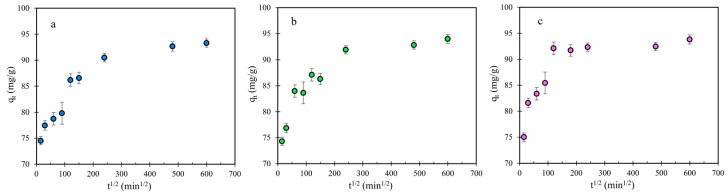
Kinetics of MB adsorption by intra-particle diffusion model under (**a**) dark, (**b**) green light, and (**c**) purple light conditions.

The effect of intra-particle diffusion was evaluated based on the curves of $q_{t}\;\mathrm{vs}.\;t^{1/2}$ (Figure 7). These curves consist of three segments, the initial curved part, boundary layer diffusion or external mass transfer [67,68], the linear part, intra-particle diffusion [65,66], and the plateau. Extrapolation of the linear portions back to the *y*-axis provides intercepts that are associated with the boundary layer thickness [67]. The lack of linearity in the linear region of the curves suggests that the intra-particle diffusion may not be the sole rate-determining step here. Furthermore, the slopes of the linear portions of the curves correspond to the intra-particle diffusion rate constants that relate to the rate of adsorption in the region controlled by intra-particle diffusion. As seen in Table 3, the intra-particle diffusion rate constant is substantially higher for the purple light conditions, where the equilibrium is achieved within the first two h (Figure 7c). Purple light is likely to accelerate the degradation of MB into smaller products, which then diffuse inside the pores at a high rate. As noted above, Figure 5c, the % degradation under purple light was higher than under green light, especially in the illumination time interval from 2 to 6 h.

The intra-particle diffusion model was also used to study the initial adsorption behavior [69]. Here $R_{i}$ value, defined as the initial adsorption factor of the intra-particle diffusion model can be calculated based on Equation (8). First, Equation (8) is rearranged in the following way

(9) $$q_{ref}=k_{i}t_{ref}{}^{1/2}+C$$

where $t_{ref}$ is the longest time in the adsorption process, and $q_{ref}$ is the amount of MB adsorbed at $t_{ref}.$ From Equation (9), $R_{i}$ can be calculated as

(10) $$R_{i}=\frac{q_{ref}-C}{q_{ref}}=1-\left(\frac{C}{q_{ref}}\right)$$

Our calculated $R_{i}$ values were ~0.2 for all conditions. As described by Wu at el. [69], $R_{i}$ values between 0.1 and 0.5 indicate strong initial adsorption. In addition, $q_{t}/q_{ref}$ at t = 30 min, was ~0.80 in all conditions, suggesting that at this point in time the initial adsorption has already reached 80%.

### 3.5. Recyclability Studies

**Figure 8:**
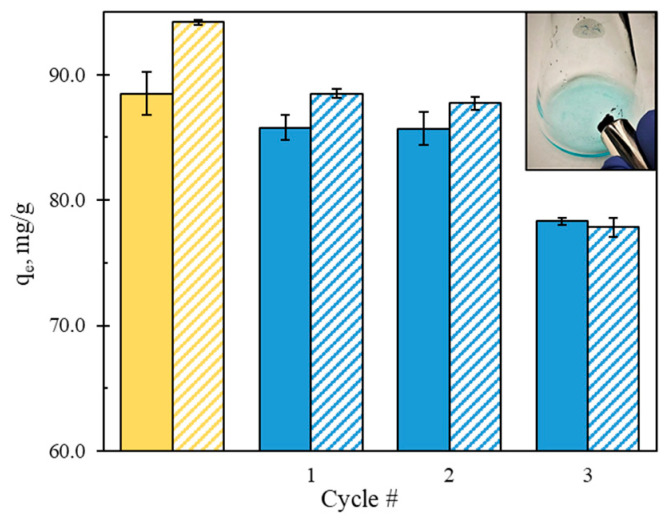
Recyclability of TPNi (solid pattern—in the dark, stripes—under purple light) and the separation of the carbon sample with a magnet. Shown in yellow are the original runs at the test conditions of pH 6, 60 mg/L MB, 600 mg/L TPNi, and 1:1:0.5 T:P:Ni ratio.

Recyclability studies were carried in the dark as described in Section 2.5. The separation of the carbon sample from the MB solution with a magnet and the results of recyclability tests are presented in Figure 8 (with the original run shown in yellow).

The sample appeared to be highly magnetic and was easily separated with the magnet. It was then reused three times after being thoroughly washed with ethanol and water after each run. As shown in Figure 8, the amount of MB removed at equilibrium decreased about 4% and 6% in the first two recyclability runs, and about 12% and 16% in the third run, in the dark and under purple light respectively. This implies the good recyclability of the TPNi sample in the dark, as well as its high photocatalytic recyclability and stability.

## 4. Conclusions

The one-pot evaporation-induced self-assembly (EISA) method was successfully applied to prepare the ordered nanoporous carbon TPNi from chestnut wood tannins, Pluronic^®^ F-127, and Ni^2+^ ions. This carbon appeared to have a 2D hexagonally ordered nanorod array mesoporous structure, with an average pore size of ~5 nm, and well dispersed nickel nanoparticles with relatively even shapes and an average particle size of 13.1 nm. Nickel nanoparticles were found to be present on the surface of OMC mainly in the form of oxides and hydroxides, with a small percentage of particles present in the reduced metal form. This TPNi carbon was then tested for removal and photocatalytic degradation of methylene blue (MB). Batch adsorption results showed that % of MB removal increased with the increase in pH and concentration of the carbon adsorbent but decreased with the increase in the concentration of MB and the increase in the mass of tannins and/or Pluronic^®^ F-127 during synthesis. Photocatalytic tests showed an increase in the amount of MB removed/degraded upon visible light illumination, where illumination with purple light at 390 nm resulted in the highest amount of MB removed, and the illumination time of ~4 h displayed the highest % degradation of MB. Moreover, the percent degradation at the lower concentration of MB was much higher than that at the higher concentration, which is likely related to the competition between MB and its photodegradation products for the active adsorption sites. The kinetics studies showed that adsorption/degradation of MB followed pseudo second-order kinetics for all testing conditions, with a strong initial adsorption, and increase in the magnitude of the rate constant from dark, to green, and purple conditions, emphasizing the positive effect of visible light on the rate of adsorption/degradation of MB. Finally, TPNi displayed good magnetic properties and recyclability, where the amount of MB removed decreased to ~12% after three rounds of recyclability.

## Figures and Tables

**Table 1:** Textural parameters calculated from nitrogen adsorption isotherms and carbonization yield of TPNi.

*S_BET_* (m^2^/g)	*V_tot_* (cm^3^/g)	*V_mic_* (cm^3^/g)	^1^ *V_mes_* (cm^3^/g)	*w* (nm)	% Yield
284	0.304	0.010	0.294	4.7	22.0 ± 1.9

^1^ Calculated from the difference between *V_tot_* and *V_mic_*.

**Table 2:** Results of the deconvolution of the XPS core-level spectra for C1s, O1s, and Ni2p, and the element content in atomic % for TPNi.

BE, eV	C1s	%	BE, eV	O1s	%	BE, eV	Ni2p	%
	C	92.73		O	6.53		Ni	0.74
284.5	C–C	75	530.5	O^2−^	21	852.5	Ni(0)	4
285.8	C–O	15	532.1	O=C/Ni(OH)_2_	53	854.5	NiO	29
287.0	C=O	5	533.7	O–C	26	856.6	Ni(OH)_2_	30
288.3	O–C=O	2				860.7	Ni 2*p*_3/2_ satellites	22
290.0	π–π*	3				863.5		14

**Table 3:** Percent degradation of MB under visible light for various types of materials.

Material	Method	Degradation %	Ref.
NiO nanoparticles	Thermal decomposition	70.2	[33]
ZnO-NiO composite	Solvothermal	85	[33]
NiO-Ag heterostructure	Hydrothermal	70	[33]
NiO-ZnO composite	Electrospinning	65.4	[33]
NiO-CuO nanoparticles	Sol-gel	40	[33]
Polymer-multiwall carbon nanotube composite	Oxidative polymerization	22–67	[58]
Carbon dot-TiO_2_ nanohybrid	Hydrothermal	16–41	[59]
Ag-doped ZnO thin films	Sol-gel	45.1	[60]
GO-hemin-TiO_2_ nanocomposite	Adsorption	90	[61]
Ceria-doped titania	Hydrothermal	85	[62]
TiO_2_-NiO heterostructures	Hydrothermal/chemical bath deposition	75–95	[63]
S-doped nanoporous carbon	Doping	55–100	[64]
S-doped NiFe catalyst	Solvothermal	73.1	[65]
**Ni-doped nanoporous carbon**	**EISA**	**84**	**this work**

**Table 4:** Psuedo first-order, pseudo second-order, and intra-particle diffusion model parameters for removal of 60 mg/L of MB by TPNi under green light, purple light, and in the dark.

	Pseudo First-Order			Pseudo Second-Order			Intra-Particle Diffusion		
	*q_e_* mg/g	*k*_1_ 1/min	R^2^	*q_e_* mg/g	*k*_2_ g/mg min	R^2^	*C* mg/g	*k*_i_ mg/g min^1/2^	R^2^
Dark	22.7	$4.2\times 10^{-3}$	0.9545	94.7	$5.0\times 10^{-4}$	0.9995	74.7	$7.1\times 10^{-2}$	0.9236
Green	22.1	$4.6\times 10^{-3}$	0.9407	95.2	$5.6\times 10^{-4}$	0.9997	76.2	$7.2\times 10^{-2}$	0.8548
Purple	18.4	$6.3\times 10^{-3}$	0.8282	94.3	$9.3\times 10^{-4}$	0.9998	74.8	$1.4\times 10^{-1}$	0.9608

## Data Availability

Not applicable.
